# Translational Venomics: Third-Generation Antivenomics of Anti-Siamese Russell’s Viper, *Daboia siamensis*, Antivenom Manufactured in Taiwan CDC’s Vaccine Center

**DOI:** 10.3390/tropicalmed3020066

**Published:** 2018-06-15

**Authors:** Libia Sanz, Sarai Quesada-Bernat, Pei Yu Chen, Cheng Dow Lee, Jen Ron Chiang, Juan J. Calvete

**Affiliations:** 1 Evolutionary and Translational Venomics Laboratory, Consejo Superior de Investigaciones Científicas (CSIC), 46010 Valencia, Spain; libia.sanz@ibv.csic.es (L.S.), squesada@ibv.csic.es (S.Q.-B.); 2 Center for Research, Diagnostics and Vaccine Development, Centers for Disease Control (CDC), 11561 Taipei, Taiwan; dow@cdc.gov.tw (C.D.L.); jrc@cdc.gov.tw (J.R.C.)

**Keywords:** *Daboia siamensis*, venomics, anti-Siamese Russell’s viper antivenom, Taiwan CDC Vaccine Center, third-generation antivenomics

## Abstract

The venom proteome of Siamese Russell’s viper from Taiwan, alongside complementary in vivo lethality neutralization assay and in vitro third-generation antivenomics assessment of the preclinical efficacy of the homologous antivenom manufactured in Taiwan CDC’s Vaccine Center, are here reported. Taiwanese Russell’s viper venom proteome comprised 25 distinct gene products, with the heterodimeric PLA_2_ viperotoxin-F representing the most abundant toxin (47.5% of total venom proteome). Coagulation FV-activating serine proteinase (RVV-V, 14%), the PIV-SVMP activator of FX (RVV-FX, 8.5%), and less abundant toxins from nine protein families, make up its venom proteome. Venom composition-pathology correlations of *D. siamensis* envenomings in Taiwan are discussed. The lethal effect of Taiwanese *D. siamensis* venom was 0.47 mg/g mouse. Antivenomics-guided assessment of the toxin recognition landscape of the Taiwanese Russell’s viper antivenom, in conjunction with complementary in vivo neutralization analysis, informed the antivenom’s maximal toxin immunorecognition ability (14 mg total venom proteins/vial), neutralization capacity (6.5 mg venom/vial), and relative content of lethality neutralizing antibodies (46.5% of the toxin-binding F(ab’)_2_ antibodies). The antivenomics analysis also revealed suboptimal aspects of the CDC-Taiwan antivenom. Strategies to improve them are suggested.

## 1. Introduction

The annual incidence of venomous snakebite in Taiwan during the period 2005–2009 was 40.49 per million persons [1]. Six of the 15 species of venomous snakes found in Taiwan are responsible for most of the clinically significant envenomations in the country [2]. These species are *Protobothrops mucrosquamatus* (Taiwan habu) (32.9% of envenomings), *Trimeresurus stejnegeri* (green habu) (24.2%), *Naja atra* (Chinese cobra) (12.1%), *Bungarus multicinctus* (Taiwan banded krait) (10.1%), *Deinagkistrodon acutus* (hundred-pace snake) (3.9%), and *D. siamensis* [3]. With a rate of 1.6% of total snakebites [3], *D. siamensis* represents the sixth most frequent cause of snakebite envenomings in Taiwan. Restricted to the south-eastern part of the country, Russell’s viper envenomings are a rare but severe medical problem [2,4,5].

Russell’s viper (Shaw and Nodder, 1797) is a terrestrial foraging snake found throughout the Indian subcontinent and much of Southeast Asia [6]. Juveniles are crepuscular foragers, feeding on lizards, whereas adults are primarily nocturnal hunters and feed mainly on rodents, especially murid species. Molecular phylogeographic studies, in relation to variation in the color pattern and symptoms of envenoming [7], identified two full species within the wide range of the Russell’s viper, *Daboia russelii* (South Asian Russell’s viper, west of the bay of Bengal, South Asia, including Bangladesh, India, Nepal, Pakistan and Sri Lanka) and *Daboia siamensis* (Siamese Russell’s viper), widely but discontinuously distributed throughout Southeast Asia, east of the Bay of Bengal, including Myanmar, Thailand, Cambodia, the Indonesian island Endeh, Flores, east Java, Komodo, and Lomblen, and southern China (Guangxi, Guangdong) and Taiwan (http://www.reptile-database.org) [8]. Russell’s vipers are highly venomous snakes. The quantity of venom produced by this heavy-bodied ambush rodent hunter is considerable, with reported venom yields for adult specimens ranging from 130–250 mg. Its LD_50_ in mice is 0.13 mg/kg intravenous, 0.40 mg/kg intraperitoneal, and 0.75 mg/kg subcutaneous [9] (http://snakedatabase.org/pages/ld50.php). For juveniles, with an average total length of 79 cm, the reported average venom yield is 8–79 mg (mean 45 mg) [10]. Lethal dose for most humans is approximately 40 to 70 mg [9]. 

Envenomings by Siamese Russell’s viper cause marked local effects, e.g., pain at the site of the bite immediately followed by swelling of the affected extremity, which may progress to tissue necrosis and a variety of systemic manifestations, including coagulopathy and hemorrhages, with bleeding from the gums, generalized increase in capillary permeability, drop in blood pressure and heart rate, rhabdomyolysis, and varied degrees of neurotoxicity, and in severe cases, disseminated intravascular coagulation leading to spontaneous bleeding from vital organs. Kidney damage, with hematuria and proteinuria, eventually triggering renal failure (attributed to poor availability or delayed administration of antivenom), have been noted frequently in Russell’s viper envenoming [11,12,13,14,15,16,17,18,19,20]. Variation in venom effects of *D. siamensis* from different geographical areas in Myanmar and Thailand have been also reported [12,13,14,15,16,17,18,19,20,21,22]. Particularities in clinical manifestations are undoubtedly functionally related to differences in venom composition across the wide geographic range of Siamese Russell’s viper. However, for this species, thus far, proteomics studies have been only conducted on venom from *D. siamensis* from Myanmar [23]. Heterodimeric PLA_2_ daboiatoxin represents the major lethal factor (LD_50_ i.p. 0.05 mg/kg body weight; crude venom, 0.6 mg/kg) of this *D. siamensis* venom, which produces neurotoxicity in mice and exhibits edema-inducing and myonecrotic activities [24].

Antivenoms to treat snakebites by the medically important venomous snake species endemic to Taiwan have been developed since the 1960s [25,26,27], including a bivalent antivenom against the hemorrhagic venoms of the green habu and the Taiwan habu; a bivalent antivenom against the neurotoxic venoms of the Taiwan banded krait and the Chinese cobra; and an antivenom against the hemorrhagic venom of the hundred-pace snake. An antivenom against the mixed type venom of the Russell’s viper (*D. siamensis*) was developed in mid-2008. Here, we have applied a venomics approach [28] to uncover the venom proteome of *D. siamensis* from Taiwan alongside in vivo and in vitro (through third-generation antivenomics) [29] assessment of the preclinical efficacy of the homologous antivenom manufactured in Taiwan CDC’s Vaccine Center. These data may contribute to enrich our knowledge on venom variations among Siamese Russell’s vipers from different geographical areas; to a better understanding of the pathogenesis of Russell’s viper envenomings in Taiwan; and to assess the toxin recognition profile of Russell’s viper antivenom that, in conjunction with in vivo neutralization analysis, may inform about its preclinical efficacy and guide initial antivenom dosage.

## 2. Materials and Methods

### 2.1. Venom and Antivenom

Venom was pooled from adult Taiwanese *D. siamensis* specimens collected in various geographic regions and maintained in captivity at the CDC (Taipei, Taiwan) for antivenom production. Pooled venom was freeze-dried and stored at −20 °C until use. Anti-Russell’s viper F(ab’)_2_ antivenom was manufactured at the Centers for Disease Control (CDC, Taipei, Taiwan) from the plasma of horses hyperimmunized with the pooled venom from Taiwanese *D. siamensis* specimens.

### 2.2. Determination of Venom LD_50_ and Antivenom ED_50_

For determination of the median lethal dose (LD_50_, the amount of venom that kills 50% of the venom-injected mice) of Taiwanese *D. siamensis* venom, groups of ten mice (weight range 11–13 g) received intraperitoneal (i.p.) injections of varying doses of venom in 0.2 mL isotonic sodium chloride solution, and the number of surviving mice in each group was recorded 48 h after venom administration. LD_50_ (mean and 95% confidence intervals, C.I.) was calculated by probit analysis of deaths occurring within 48 h of venom injection [30]. For determination of the antivenom’s median effective dose (ED_50_, the least amount of antivenom required to prevent death in 50% of mice injected with n× LD_50_s of venom), one vial of anti-*Daboia siamensis* antivenom (156 mg F(ab’)_2_; expiry date 30 October 2019) was reconstituted in 10 mL of supplied diluent and variable doses were mixed with eight venom LD_50_s, the final volume was made up to 0.2 mL isotonic sodium chloride solution, and the mixture was incubated at 37 °C for 60 min. The mixtures were i.p. injected in ICR mice (n = 10 per group, weight range 11–13 g) and 48 h later the number of surviving mice in each group was recorded. The median effective dose (ED_50_) and 95% C.I. was calculated by probit analysis of deaths occurring within 48 h of venom injection [30]. All procedures used in this study were approved by the Institutional Animal Care and Use Committee (IACUC) of CDC Taiwan (approval number 10601) and meet the international guiding principles for biomedical research involving animals.

### 2.3. Isolation and Initial Characterization of D. siamensis Venom Proteins

Venom of Taiwanese *D. siamensis* was analyzed using the previously-described venomics workflow [31]. Briefly, two milligrams of crude lyophilized venom were dissolved in 300 μL of 0.05% trifluoroacetic acid (TFA) and 5% acetonitrile (ACN). Insoluble material was removed by centrifugation in an Eppendorf centrifuge at 13,000× *g* for 10 min at room temperature, and the proteins contained in 15 μL were separated by reverse-phase (RP)-HPLC using a Agilent LC 1100 High Pressure Gradient System equipped with diode array detector (DAD) and a Discovery^®^ BIO Wide Pore C18 (15 cm × 2.1 mm, 3 μm particle size, 300 Å pore size) column (St. Louis, MO, USA). The column was developed at a flow rate of 0.4 mL/min with a linear gradient of 0.1% TFA in MilliQ^®^ water (Merck-Millipore, Darmstadt, Germany) (solution A) and 0.1% TFA in acetonitrile (solution B), isocratic (5% B) for 1 min, followed by 5–25% B for 5 min, 25–45% B for 35 min, and 45–70% B for 5 min. Protein detection was carried out at 215 nm with a reference wavelength of 400 nm. Fractions were collected manually across the entire elution range, dried in a vacuum centrifuge (Savant), and redissolved in MilliQ^®^ water. Molecular masses of the purified proteins were estimated by non-reduced and reduced SDS-PAGE (on 12 or 15% polyacrylamide gels), or determined by electrospray ionization (ESI) mass spectrometry. For SDS-PAGE analysis, sample aliquots were mixed with ¼ volume of 4× sample buffer (0.25 M Tris-HCl pH 6.8, 8% SDS, 30% glycerol, 0.02% bromophenol blue, with or without 10% 2-mercaptoethanol) and heated at 85 °C for 15 min, run under non-reducing and reducing conditions, and the gels stained with Coomassie Brilliant Blue G-250. For ESI-MS, the proteins eluted in the different RP-HPLC fractions were separated by nano-Acquity UltraPerformance LC^®^ (UPLC^®^) using a BEH130 C18 (100 µm × 100 mm, 1.7 µm particle size) column (Waters Corp., Milford, MA, USA) in-line with a Waters SYNAPT G2 high definition mass spectrometry system. The flow rate was set to 0.6 µL/min and the column was developed with a linear gradient of 0.1% formic acid in water (solution A) and 0.1% formic acid in ACN (solution B), isocratically 1% B for 1 min, followed by 1–12% B for 1 min, 12–40% B for 15 min, 40–85% B for 2 min. Monoisotopic and isotope-averaged molecular masses were calculated by manually deconvolution of the isotope-resolved multiply-charged MS1 mass spectra.

### 2.4. Venomics Characterization and Quantification of the Venom Proteome of Taiwanese Russell’s Viper

Protein bands were excised from Coomassie Brilliant Blue-stained SDS-PAGE gels and subject to in-gel reduction (10 mM dithiothreitol, 30 min at 65 °C) and alkylation (50 mM iodacetamide, 2 h in the dark at room temperature), followed by overnight sequencing-grade trypsin digestion (66 ng/μL in 25 mM ammonium bicarbonate, 10% ACN; 0.25 μg/sample) in an automated processor (ProGest Protein Digestion Workstation, Genomic Solution Ltd., Cambridgeshire, UK). Tryptic digests were dried in a vacuum centrifuge (SPD SpeedVac^®^, Thermo Scientific Inc., West Palm Beach, FL, USA), re-dissolved in 15 μL of 5% ACN containing 0.1% formic acid, and submitted to LC-MS/MS. Tryptic peptides were separated by nano-Acquity UltraPerformance LC^®^ (UPLC^®^), as above. Doubly- and triply-charged ions were selected for CID-MS/MS. Fragmentation spectra were interpreted (i) manually (*de novo* sequencing), (ii) using the on-line form of the MASCOT Server (version 2.6) at http://www.matrixscience.com against the last update (release 218, 15 February 2017) of NCBI non-redundant database, and iii) processed in Waters Corporation’s ProteinLynx Global SERVER 2013 version 2.5.2. (with Expression version 2.0). The following search parameters were used: Taxonomy: all entries; Enzyme: trypsin (1 missed cleavage allowed); MS/MS mass tolerance was set to ±0.6 Da; carbamidomethyl cysteine and oxidation of methionine were selected as fixed and variable modifications, respectively. All matched MS/MS data were manually checked. Peptide sequences assigned by *de novo* MS/MS were matched to homologous proteins available in the NCBI non-redundant protein sequences database using the online BLASTP program [32] at https://blast.ncbi.nlm.nih.gov/Blast.cgi. The relative abundances of the chromatographic peaks obtained by reverse-phase HPLC fractionation of the whole venom were calculated as ‘% of total peptide bond concentration in the peak’ by dividing the peak area by the total area of the chromatogram [31,33]. For chromatographic peaks containing single components (as judged by SDS-PAGE and/or MS), this figure is a good estimate of the % by weight (g/100 g) of the pure venom component [34]. When more than one venom protein was present in a reverse-phase fraction, their proportions (% of total protein bands area) were estimated by densitometry of Coomassie-stained SDS-polyacrylamide gels using MetaMorph^®^ image analysis software (Molecular Devices, San Jose, CA, USA). Conversely, the relative abundances of different proteins contained in the same SDS-PAGE band were estimated based on the relative intensities of the three most abundant peptide ions associated with each co-migrating protein by MS/MS analysis. The relative abundances of the protein families present in the venom were calculated as the ratio of the sum of the percentages of the individual proteins from the same family to the total area of venom protein peaks in the reverse-phase chromatogram. 

### 2.5. Two-Dimensional (IEF/SDS-PAGE) Gel Electrophoresis (2-DE)

2-DE was performed essentially according to the manufacturer’s (GE Healthcare Amersham Biosciences) instructions, unless otherwise indicated. For isoelectric focusing (IEF), ~150 μg of venom was dissolved in 7 M urea, 2 M thiourea, 4% CHAPS, (with or without 40 mM DTT), and 0.5% IPG buffer pH 3–10 and applied onto 7-cm pH 3–10 linear immobilized pH gradient (IPG) strips. IEF was carried out with an Ettan-IPGphor isoelectric focusing unit at 20°C applying the following conditions: 300 V (0.5 h), ramping to 1000 V (0.5 h), ramping to 5000 (1.3 h) and 5000 V (0.5 h). After IEF, the IPG strips were kept at −70°C until use. For the second (SDS-PAGE) dimension, the IPGs were equilibrated for 15 min with gentle shaking at room temperature in equilibration buffer (6 M urea, 2% (*w*/*v*) SDS, 30% (*v*/*v*) glycerol, 75 mM Tris–HCl (pH 8.8), with or without 40 mM DTT). IPG strips were then placed on top of an SDS-15% polyacrylamide gels and run in a Protean II (Bio-Rad, Hercules, CA, USA) electrophoresis unit at room temperature. Protein spots were visualized by staining using Coomassie Brilliant Blue G250 (Sigma-Aldrich, St. Louis, MO, USA).

### 2.6. Third-Generation Antivenomics

Third-generation antivenomics [29] was applied to assess the immunoreactivity of the CDC antivenom against venom from *D. siamensis* from Taiwan. One vial of antivenom was dissolved in 10 mL of the supplied diluent, dialyzed against MilliQ^®^ water, lyophilized, and reconstituted in 10 mL of 0.2 M NaHCO_3_, 0.5 M NaCl, pH 8.3 (coupling buffer). The concentrations of the antivenom stock solution was determined spectrophotometrically using an extinction coefficient for a 1 mg/mL concentration (ε^0.1%^) at 280 nm of 1.36 (mg/mL)^−1^ cm^−1^ [35]. Antivenom affinity columns were prepared in batches. Three mL of CNBr-activated Sepharose™ 4B matrix (Ge Healthcare, Buckinghamshire, UK) were packed in a ABT column (Agarose Bead Technologies, Torrejón de Ardoz, Madrid, Spain) and washed with 10× matrix volumes of cold 1 mM HCl, followed by two matrix volumes of coupling buffer to adjust the pH to 7.0–8.0. CNBr-activated instead of *N*-hydroxysuccinimide (NHS)-activated matrix was employed because NHS released during the coupling procedure absorbs strongly at 280 nm, thus interfering with the measurement of the concentration of antibodies remaining in the supernatant of the coupling solution. Forty milligrams of antivenom was dissolved in 2× matrix volume of coupling buffer and incubated with 3 mL of CNBr-activated matrix for 4 h at room temperature. Antivenom coupling yield, estimated measuring A_280nm_ before and after incubation with the matrix, was 23.1 mg/mL. After the coupling, remaining active matrix groups were blocked with 12 mL of 0.1 M Tris-HCl, pH 8.5 at room temperature for 4 h. Affinity columns, each containing 300 µL (7 mg) of immobilized antivenom, were alternately washed with three matrix volumes of 0.1 M acetate containing 0.5 M NaCl, pH 4.0–5.0, and three matrix volumes of 0.1 M Tris-HCl, pH 8.5. This procedure was repeated six times. The columns were then equilibrated with five volumes of working buffer (PBS, 20 mM phosphate buffer, 135 mM NaCl, pH 7.4) and incubated with increasing amounts (100–1200 μg of total venom proteins) of *D. siamensis* (Taiwan) dissolved in ½ matrix volume of PBS, and the mixtures incubated for 1 h at 25 °C in an orbital shaker. As specificity controls, 300 μL CNBr-activated Sepharose™ 4B matrix, without (mock) or with 8 mg of immobilized control (naïve) horse IgGs, were incubated with venom and developed in parallel to the immunoaffinity columns. The non-retained eluates of columns incubated with 100–600 μg and 900–1500 μg venom were recovered with 5× and 10× matrix volume of PBS, respectively, and the immunocaptured proteins were eluted, respectively, with 5× and 10× matrix volume of 0.1 M glycine-HCl, pH 2.7 buffer and brought to neutral pH with 1 M Tris-HCl, pH 9.0. The entire fractions eluted with 5× and ½ of the fractions recovered in 10× matrix volume were concentrated in a Savant SpeedVac™ vacuum centrifuge (ThermoFisher Scientific, Waltham, MA, USA) to 40 μL, and aliquots corresponding to 150 initial total venom proteins were fractionated by reverse-phase HPLC using an Agilent LC 1100 High Pressure Gradient System (Santa Clara, CA, USA) equipped with a Discovery^®^ BIO Wide Pore C18 (15 cm × 2.1 mm, 3 μm particle size, 300 Å pore size) column and a DAD detector as above. Eluate was monitored at 215 nm with a reference wavelength of 400 nm. The fraction of non-immunocaptured molecules was estimated as the relative ratio of the chromatographic areas of the toxin recovered in the non-retained (NR) and retained (R) affinity chromatography fractions using the equation %NRi = 100 − [(Ri/(Ri + NRi)) × 100], where Ri corresponds to the area of the same protein ‘i’ in the chromatogram of the fraction retained and eluted from the affinity column.

## 3. Results and Discussion

### 3.1. The Venom Proteome of Siamese Russell’s Viper from Taiwan

**Figure 1:**
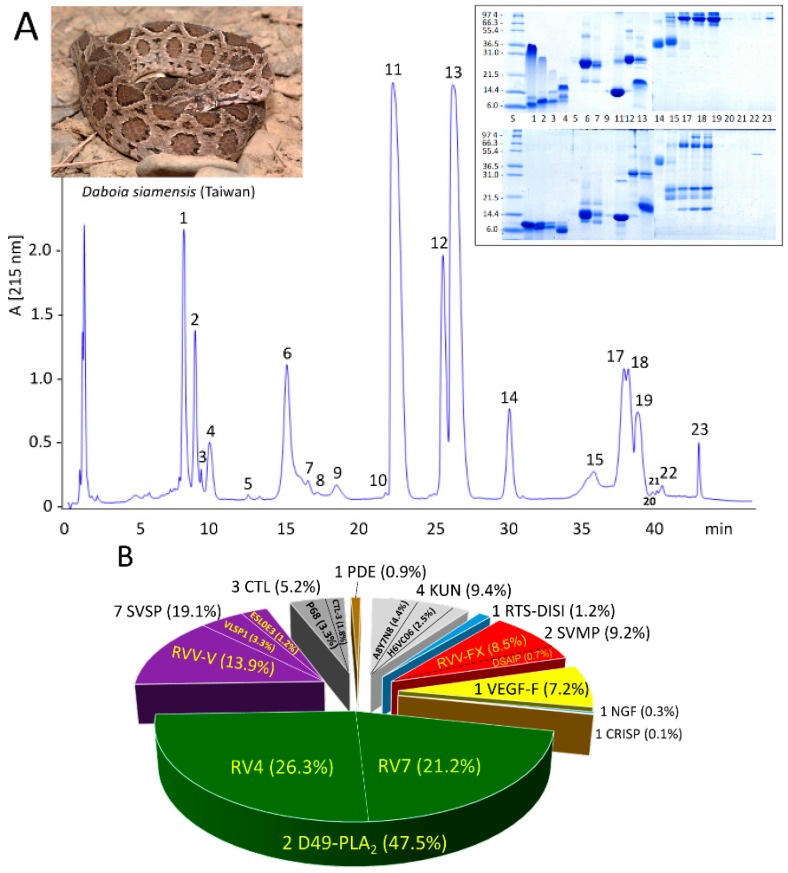
Venomics analysis of *D. siamensis* from Taiwan. Panel (**A**), reverse-phase chromatographic separation of the venom proteins. Chromatographic fractions were collected manually and analyzed by SDS-PAGE (inset) under non-reduced (upper panels) and reduced (lower panels) conditions. Protein bands were excised, in-gel digested with trypsin, and the resulting tryptic peptides sequenced by LC-nESI-MS/MS and identified by database searching or *de novo* sequencing (Supplementary Materials Table S1). Panel (**B**), pie chart displaying the number and relative occurrence (in percentage of total venom proteins) of toxins from different protein families identified in the venom proteome (listed in Table 1). Acronyms: D49-PLA_2_, D49 phospholipase A_2_; SVSP, snake venom serine protease; CTL, C-type lectin-like; PDE, phosphodiesterase; KUN, Kunitz-type serine proteinase inhibitor-like protein; RTS-DISI, RTS-disintegrin; SVMP, snake venom metalloptoteinase; VEGF, vascular endothelial growth factor; NGF, nerve growth factor; CRISP, cysteine-rich secretory protein.

**Figure 2:**
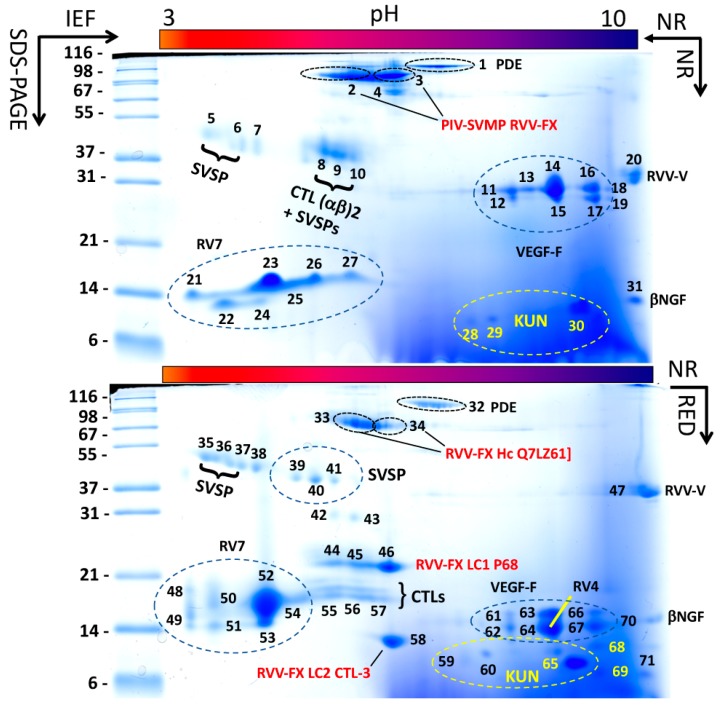
Two-dimensional electrophoretic reference maps of *D. siamensis* (Taiwan) venom. Upper and lower panels display, respectively, two-dimensional electrophoretic separations of ~150 μg of venom proteins run under non-reducing (NR) conditions in both dimensions, and under NR condition in the first dimension (IEF) and reducing (RED) conditions in the second (SDS-PAGE) dimension. MS/MS assignments of protein spots are listed in Supplementary Materials Table S2. This approach revealed that CTL P68 (spot 46) and CTL-3 (spot 58) were covalently linked to SVMP [Q7LZ61] (spot 34) forming a structural part of the PIV-SVMP RVV-FX (spot 3). Spots of other venom proteins, e.g., the PLA_2_ subunits of the RV4/RV7 heterodimeric complex, SVSP activator of Factor V (RVV-V), VEGF-F, βNGF, dimeric CTLs, PDE, SVSP, KUN, are also labeled. For more details, please consult Supplementary Materials Table S2.

*D. siamensis* (Taiwan) venom was fractionated by reverse-phase (RP) HPLC (Figure 1A) and the RP fractions analyzed by SDS-PAGE (Figure 1A, inset). Venom proteins were also separated by two-dimensional (2DE) electrophoresis (IEF/SDS-PAGE) run under non-reducing conditions in both directions and under non-reducing IEF, followed by reduced SDS-PAGE conditions (Figure 2). SDS-PAGE- and 2DE-resolved protein bands were identified via a tryptic-peptide-centric MS/MS approach and database matching through MASCOT search engine or BLAST analysis of *de novo* sequenced peptide ions (Supplementary Materials Table S1). Figure 1B displays the relative abundances (in percentage of total venom proteins) of the ten different protein families identified, highlighting the identity or closest homolog of major venom components.

The venom proteome of *D. siamensis* (Taiwan) comprises 25 distinct gene products (Table 1), including seven snake venom serine proteinases (SVSP), four Kunitz-type serine proteinase inhibitor-like (KUN) molecules; five C-type lectin-like (CTL) proteins; two snake venom metalloproteinases (SVMP); two phospholipase A_2_ (PLA_2_) molecules, and one protein species of each RTS-disintegrin (DISI), vascular endothelial growth factor (VEGF), nerve growth factor (NGF), cysteine-rich secretory protein (CRISP), and phosphodiesterase (PDE) protein families (Table 1). 

Analysis of the venom by 2DE revealed that some of these proteins, particularly PLA_2_s and CTLs, formed a structural part of macromolecular complexes. Thus, the two PLA_2_ molecules, RV4 and RV7, associated into a non-covalent heterodimer, which accounted for 47.5% of the total venom proteome (Figure 1B) (PDB accession code 1OQS) [36]. CTL-3, CTL-4, and P68 formed disulphide-bonded complexes between themselves. In addition, CTL-3 and P68, along with CTLs Q4PRD1 and ADJ67473, were found engaged in covalent (S-S) association with PIII-SVMP Q7LZ61 into the heterotrimeric PIV-metalloproteinase RVV-X (PDB 2E3X) (Figure 2; Table S2) [37].

### 3.2. Comparison of the Venom Proteomes of D. siamensis from Taiwan and Myanmar

*D. siamensis* (Myanmar) venom proteome was previously investigated by 2DE, and the separated venom spots were subjected to in-gel digestion and identification by LC-MS/MS of tryptic peptides [23]. The 2DE separation patterns of the *D. siamensis* venoms from Taiwan and Myanmar looked very similar (compare Figure 1 of [23] and Figure 2 of this work). Like the venom proteome of Taiwanese *D. siamensis*, the 2DE-separated toxins from *D. siamensis* from Myanmar were assigned [23] to serine proteases, including RVV-FV proteoforms, thrombin-like and β-fibrinogenase; isoforms of the heavy and light chains of FX activating PIV metalloprotease, RVV-FX; acidic and basic phospholipases A_2_, including the acidic chain of the RV4/RV7 complex; l-amino acid oxidases; vascular endothelial growth factors (VEGFs); and C-type lectins. Unlike in Taiwanese *D. siamensis* venom, neither PDE, cysteine-rich secretory proteins (CRISPs), NGF, KUN, or disintegrins were identified. However, the presence of low molecular mass (less than 10 kDa, e.g., disintegrins and KUN) cannot be excluded because only proteins of more than 10 kDa were resolved in the 2DE gel of Myanmar *D. siamensis* venom [23].

### 3.3. Functional Correlations

Like clinical features recorded in Siamese Russell’s viper envenomings in other South East Asian regions, incoagulable blood resulting from consumption coagulopathy [38], with bleeding diathesis and hemolysis leading to spontaneous bleeding from vital organs (kidney, blood, muscle, brain and gastrointestinal tract), represent the major systemic symptoms found in envenomings of patients bitten from 1987 to 1999 by this species in the south-eastern regions of Taiwan [14]. Systemic thrombosis, manifested as multiple cerebral infarctions, seems to be a distinguishing feature of Taiwanese Russell’s viper snakebites [14]. *D. siamensis* (Taiwan) venom is specially endowed with a battery of proteins, of which the toxic activity converges in impairing through different mechanisms the victim/prey’s capability to control blood clotting or coagulation [39].

Massive thrombosis of large vessels followed by death within a few minutes occurs in the natural prey of Russell’s viper snakes [40]. Although in human envenomings the dose of venom injected is insufficient to cause massive fatal intravascular coagulation in the large vessels and the heart [40], there is systemic deposition of microthrombi in medium and small vessels, often inducing multiorgan (heart, lung, central nervous system, and kidney) damage, with irreversible ischemic changes leading to morbidity and death. This pathological picture underlies the devastating hematological effects of the venoms of Russell’s vipers across their entire geographical distribution [7,41,42]. RVV-FX and RVV-V, abundant proteins of Taiwanese Russell’s viper venom (Figure 1B), represent major players of this hemostatic imbalance. RVV-X (8.5% of Taiwanese *D. siamensis* venom proteome) specifically, activates coagulation Stuart factor (factor X, FX) by cleaving the same Arg-Ile bond that is cleaved by factors IXa and VIIa during physiological coagulation [43,44]. Thrombin-like serine protease RVV-V (14% of *D. siamensis* (Taiwan) venom proteins), specifically activates factor V by the selective single cleavage at site III (Arg1545-Ser1546) [45]. The structural basis of this narrow substrate specificity has been solved by X-ray crystallography [46]. Factors Va and Xa assemble on the membrane of platelets into the prothrombinase complex that catalyses the formation of α-thrombin, initiating several positive-feedback reactions that sustain its own formation and facilitate the formation and dissemination of microthrombi in the microcirculation, with consumption of FX, FV, fibrinogen and platelets resulting in the development of life-threatening disseminated intravascular coagulation.

Venom of *D. siamensis* from Taiwan has been reported to be neurotoxic, less haemolytic, and weakly myonecrotic in animals [47]. However, contrary to the results in animals, no neurotoxic manifestations, such as facial palsy, ptosis, external ophthalmoplegia, and facial weakness, which have been reported to be a prominent feature of Russell’s viper bite in Sri Lanka and South India [40,48], are not observed in human envenomings by Siamese Russell’s viper in Taiwan [14]. The clinically neurotoxic manifestation might be the most important difference between symptoms of snakebite by western Russell’s viper compared to the eastern type. Neurotoxicity following Sri Lankan Russell’s viper envenoming is primarily due to the pre-synaptic neurotoxin U1-viperitoxin-Dr1a, a major monomeric PLA_2_ molecule [P86368] (13672.82 Da) that constitutes 19.2% of the crude venom [49,50]. On the other hand, presynaptic viperotoxin F, a heterodimer of two highly homologous (65% identity) but oppositely charged PLA_2_ subunits, a basic and neurotoxic (RV-4) [Q02471] subunit and an acidic non-toxic component (RV-7) [P31100] with a very low enzymatic activity [36], has been identified as the most abundant (47.5% of the venom proteins, Figure 1B) and lethal (to mice) venom component of Taiwanese *D. siamensis* [51].

Elucidating the mechanisms by which viperotoxin F causes different toxic effects in mice and humans requires detailed studies. However, it is worth noting that crotoxin, another heterodimeric PLA_2_ molecule and the major component of the venom of juvenile Central American rattlesnake, *Crotalussimus*, and juvenile and adult South American rattlesnake, *C. durissus terrificus* [52], like viperitoxin F, exhibits potent (LD_50_: 0.07 (C.I. 95%: 0.06–0.09) μg/g mouse) presynaptic β-neurotoxicity in the murine natural prey [53,54,55,56,57,58]. However, human envenomings by juvenile *C. simus* develop myotoxic and coagulant effects without apparent neurological manifestations [54,59,60,61]. Envenomations by South American rattlesnakes present systemic neuro- and myotoxicity, myalgic symptoms, and disseminated intravascular coagulation (DIC), with frequent renal failure accompanied by acute tubular necrosis [62,63]. Hence, the toxic phospholipase activity of Taiwanese Russell’s viper venom might contribute, as suggested previously [64,65], to the microangiopathic hemolysis associated with DIC.

Other venom components that likely contribute to the haemostatic disturbance in victims of Siamese *D. russelii* envenoming include the Kunitz-type serine protease inhibitor-like (KUN) proteins. Although the biological activities of most KUN present in Russell’s viper venoms are poorly defined, rusvikunin-I and rusvikunin-II present in the venom of *D. russelii* from Pakistan exhibited in vitro trypsin, plasmin, and thrombin inhibitory activity via a non-enzymatic mechanism, and in vivo potent anticoagulation effect [66]. DrKIn-II [H6VC06], a major KUN protein in Taiwanese *D. siamensis* (Figure 1B), dose-dependently inhibits plasmin (Ki 0.19 nM) [67], and DrKIn-I potently block activated protein C (Ki 53 pM) [68], exacerbating hypofibrinogenemia induced by RVV-X and potentiating consumptive coagulopathy in Russell’s viper envenomation [68].

Snake venom C-type lectin-like molecules (snaclecs) target a wide range of coagulation factors, other proteins critical in hemostasis, including membrane receptors on platelets displaying opposite functions on blood coasgulation and platelet aggregation/agglutination [69,70]. The pathophysiological relevance of the CTL proteins identified in the venom of Taiwanese *D. siamensis* (CTL-3, CTL-4, and P68) (Table 1) has not been studied. However, other *Daboia* CTLs impaired ristocetin-induced platelet agglutination [71] or exhibit anticoagulant activity through uncompetitive inhibition of FXa [72], contributing to the aggravation of the venom-induced consumptive coagulopathy.

Venom phosphodiesterase is also likely to contribute to the haemostatic impairment in victims of *D. siamensis* envenoming. This enzyme hydrolyses ADP, strongly inhibiting ADP-induced platelet aggregation in human platelet-rich plasma [73]. In addition, it has been suggested that the disturbance in extracellular nucleotides and purines levels generated from endogenous precursors may potentiate venom-induced hypotension and paralysis via purine receptors [74]. Snake venom VEGF proteins increase vascular permeability [75], thereby acting as an agent of toxin dispersion, which may also contribute to the hypotensive action of the venom.

### 3.4. In Vivo and In Vitro Analysis of the Preclinical Efficacy of Anti-Daboia siamensis (Taiwan) Antivenom

For patients who survived the first 24–48 h after envenoning by Taiwanese Russell’s viper, renal failure due to massive occlusion of the renal microvasculature and parenchyma ischemia [41] emerged as one of the most devastating effects of envenomation. The timely administration of an effective antivenom is the only scientifically-validated therapeutic treatment of snakebite envenoming. To assess the immunological profile of the anti-*Daboia siamensis* antivenom manufactured in Taiwan CDC’s Vaccine Center, we have investigated its capability to reverse venom lethality in mice and its toxin-resolved immunorecognition landscape.

#### 3.4.1. Venom Lethality and Antivenom Neutralization of the Venom’s Lethal Effect

The lethal effects (expressed as LD_50_) of Taiwanese *D. siamensis* (Ds) venom to mice was 5.68 µg/11–13 g mouse (0.47 µg/g mouse). Using a challenge dose of 8× LD_50_, the CDC-Taiwan antivenom (AV) showed a potency of 131 units/mL (1310 U/vial), which corresponds to an ED_50_ of 10.27 (C.I. 95%: 7.95–12.67) mg Ds venom/g F(ab’)_2_, or a potency P ([mg venom neutralized/mL antivenom] = [(Tν − 1)/ED_50_] × LD_50_, where Tν is the number of LD_50_ used as challenge dose, and ED_50_ is expressed as mL antivenom that protect 50% of the inoculated mice population) of 6.5 mg venom/vial (156 mg F(ab’)_2_).

#### 3.4.2. Assessment of the Immunorecognition Landscape of Taiwanese Anti-D. siamensis Antivenom through Third-Generation (3G) Antivenomics

**Figure 3:**
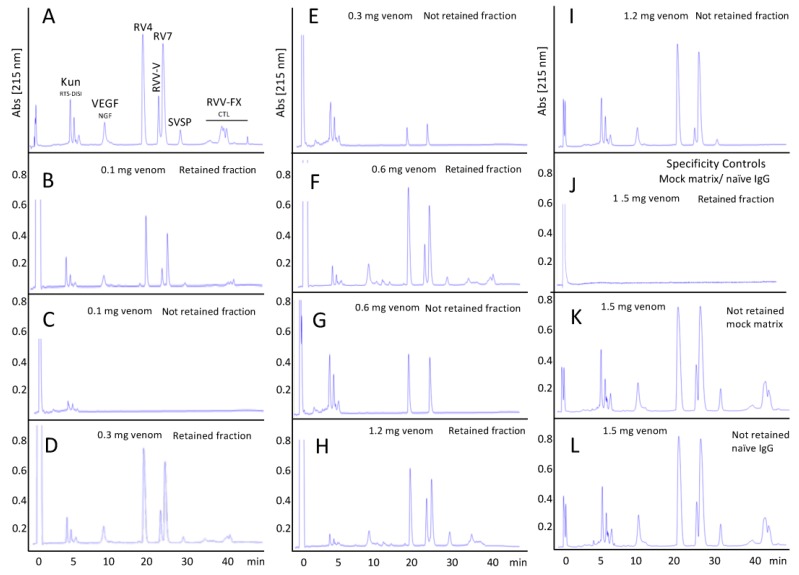
Third-generation antivenomics of CDC anti-*Daboia siamensis* antivenom. Reverse-phase chromatographic analysis of whole venom (panel (**A**)) and of the non-retained and the immunoretained fractions recovered from affinity column (7 mg immobilized CDC-Taiwan anti-*Daboia siamensis* antivenom F(ab’)_2_ molecules) incubated with increasing amounts (100–1200 μg) of *D. siamensis* venom (panels (**B**–**I**)). Panels (**J**–**L**), retained and non-retained venom fractions on mock matrix and naïve equine IgG affinity columns.

The ability of the CDC antivenom to recognize the toxins present in the venom of the Siamese Russell’s viper from Taiwan was assessed through affinity chromatography-based 3G antivenomics [29], using CDC antivenom immunoaffinity columns to quantitate the immunocapturing capability of the immobilized antivenom towards each of a venom’s reverse-phase separated chromatographic fractions. To this end, identical immunoaffinity columns were run in parallel using increasing amounts of venom until saturation was reached. Figure 3 displays reverse-phase chromatographic separations of the non-retained and the immunoretained fractions recovered from affinity column (7 mg immobilized antivenom F(ab’)_2_ molecules) incubated with increasing amounts (100–1200 μg) of *D. siamensis* venom. Table 2 shows the capacity of the immobilized antivenom’s F(ab’)_2_ molecules to bind the major Taiwanese *D. siamensis* venom components as a function of the concentration of the incubated venom. The data listed in Table 2 indicate that the CDC anti-*Daboia siamensis* antivenom has a good capacity to recognize its major venom cognate toxins, which, at the concentration of venom that causes maximal binding, ranges from 27–43% of KUN molecules to 59–80% of PLA_2_s and SVSPs, and 100% of VEGF/NGF, CTLs and RVV-FX. The antivenomics data indicate that the antivenom has the capacity to bind 627.7 μg of venom proteins per immunoaffinity column, or in other words, 89.7 mg total venom proteins/g F(ab’)_2_, or 14 mg venom/vial containing 156 mg F(ab’)_2_. Assuming that the ratio between the antivenom’s potency (V_neutralized_) (e.g., the amount of venom the antivenom is capable of neutralizing) and the amount of venom that the antivenom is capable of binding (V_bound_) represents a proxy for the fraction of therapeutic antibodies of that antivenom, the calculated fraction of active toxin-binding F(ab’)2 molecules [(V_neutralized_)/(V_bound_)] of the CDC-Taiwan anti-Ds antivenom equals 6.5/14 = 46.4%.

#### 3.4.3. Functional Comparison of the CDC Antivenom with Other Antivenoms against Russell’s Viper Venoms

The combination of maximal binding capacity and potency are relevant functional characteristics of an antivenom that allow comparison of antivenoms produced by different manufacturers. Thus, the maximal venom toxins binding capacity of the CDC-Taiwan anti-*D. siamensis* antivenom (89.7 mg total venom proteins/g F(ab’)_2_) compares favorably with the same parameter of other commercial antivenoms manufactured in India against homolog *D. russelii* venom, which ranges between 17–39 mg venom/g F(ab’)_2_ [76]. However, the Indian products contain about 500 mg F(ab’)_2_ per vial vs. 156 mg F(ab’)_2_/vial of the Taiwanese antivenom, and thus, on a vial basis, both antivenoms have similar binding characteristics: 14 (Taiwan) versus 8.6–19.6 (India) mg venom/vial. The nominal specifications of the Indian antivenoms indicate that ‘1 mL neutralises not less than 0.6 mg of cobra (*N. naja*) venom, 0.45 mg of common krait (*B. caeruleus*) venom, 0.6 mg of Russell’s viper (*D. russelii*) venom, and 0.45 mg of saw-scaled viper (*E. carinatus*) venom’. This neutralization capacity (6 mg *D. russelii* venom per vial) is similar to the potency determined for the CDC-Taiwan antivenom (6.5 mg *D. siamensis* venom/vial), but much better than the potency of Hemato Polyvalent Snake Antivenom (HPSA) (against *Cryptelytrops albolabris*, *Calleoselasma rhodostoma*, and *D. siamensis*, all of Thai origin) and monospecific (DS, LD_50_: 0.49 (C.I. 95%, 0.40–0.60) μg/g mouse) produced at QSMI (Queen Saovabha Memorial Institute, Bangkok, Thailand) [77]. These Thai antivenoms were produced in horses immunized using the ‘low dose, low volume multi-site’ immunization protocol until the antisera showed rapid rise in ELISA titers and reached plateau at about the 8^th^ week post-immunization. The in vivo neutralization potency (P) of the antisera against *C. albolabris*, *C. rhodostoma*, and *D. siamensis* venoms was 10.40, 2.42, and 0.76 mg/mL, respectively, and the corresponding potency for the QSMI monospecific antiserum against *D. siamensis* venom was 1.50 mg/mL (ED_50_: 1.88 (1.53–2.30) mg/mL) [76]. The Thai antisera were effective in neutralizing the nephrotoxicity of the homologous *D. siamensis* venom, and the HPSA (19.82 ± 1.39 g F(ab’)_2_/L) antivenom also cross-neutralized other common Southeast Asian viperid venoms, including *D. siamensis* (Myanmar) (LD_50_: 0.34 (0.08–0.81) mg/g mouse) with ED_50_ of 5.00 (2.38–11.0) mg/mL, and potency of 4.00 mg/mL [78].

## 4. Conclusions

The Indian polyspecific antivenoms have a relative proportion of neutralizing toxin-binding antibodies of 30–50%, while in the Taiwanese antivenom 46.5% of the toxin-binding F(ab’)_2_ antibodies are lethality-neutralizing molecules. These figures indicate that the Taiwan antivenom could be substantially improved (up to a potency of 20.8 mg venom/vial) by simply adjusting the amount of F(ab’)_2_ molecules per vial to the same level (500 mg/vial) as many other commercial antivenoms, including the Indian polyvalent products. This measure would result in a significant reduction in the number of vials needed for a snakebite treatment, with the consequent reduction in the risk of causing adverse reactions. 

South and Southeast Asian Siamese Russell’s viper venoms contain toxins that act synergistically to consume and deplete circulating blood clotting factors, resulting primarily in hemostatic disturbances promoting disseminated intravascular coagulation and systemic hemorrhage following envenoming, and secondary life-threatening acute kidney injury (AKI) [79,80,81]. Increasing the absolute potency per vial of the CDC monospecific antivenom would presumably contribute to treat more effectively severe envenomings by *D. siamensis* in Taiwan. Given its good immunological profile, it would be advisable to conduct preclinical studies of the paraspecific effectiveness of the CDC antivenom towards geographic variants of *D. siamensis* venom.

## Figures and Tables

**Table 1:** Summary of the proteins that make up the venom proteome of Siamese Russell’s viper from Taiwan. For details of venomics assignments, consult Supplementary Materials Table S1.

	Protein			Species
P18964	SVSP	RVV-V alpha	FV activator	*D. siamensis*
P18965	SVSP	RVV-V gamma		*D. siamensis*
P86530	SVSP	RVV-V homolog-1		*D. russelii*
P86531/2	SVSP	RVV-V homolog-4		*D. russelii*
E0Y418	SVSP	VLSP-1		*M. lebetina*
E0Y419	SVSP	VLBF	β-fibrinogenase	*M. lebetina*
ESL0E3	SVSP	VLAF	α-fibrinogenase	*D. siamensis*
A8Y7N8	KUN	KUN-5		*D. siamensis*
H6VC06	KUN	DrK-In-II		*D. russelii*
AFE83617	KUN			*D. siamensis*
A8Y7N6/7	KUN	KUN-3/4		*D. siamensis*
ADK22825	CTL	P68 a-subunit	Light chains of PIV-SVMP RVV-FX (αβ)n, n ≥ 2	*D. siamensis*
Q4PRD0	CTL	C-type lectin-3		*D. siamensis*
Q4PRC9	CTL	C-type lectin-4		*D. siamensis*
Q4PRD1	CTL	RVV-FX Light Chain-1	PIV-SVMP FX activator	*D. siamensis*
ADJ67473	CTL	RVV-FX Light Chain-2		*D. siamensis*
Q7LZ61	SVMP	RVV-FX Heavy Chain		*D. siamensis*
AUF41660	SVMP	DSAIP	PIII-SVMP Daborhagin-K	*D. siamensis*
Q02471	PLA2	Basic subunit RV4	Viperotoxin-F	*D. siamensis*
P31100	PLA2	Acidic subunit RV7		*D. siamensis*
AUF41658	DISI	RTS-disintegrin	Russelistatin	*D. siamensis*
P0DL42	VEGF	VEGF-F		*D. siamensis*
P30894	NGF			*D. russelii*
ACE73567	CRISP			*D. russelii*
AHJ80885	PDE			*M. lebetina*

**Table 2:** Total and concentration-dependent immunoretained (RET) *D. siamensis* (Taiwan) venom proteins by CDC antivenom affinity columns containing 7 mg of immobilized F(ab’)_2_ molecules. Maximal binding for each RP-HPLC fraction is highlighted in yellow background.

RP-HPLC Fraction		*Daboia siamensis* (Taiwan) Total Venom Proteins (mg)						Major Toxins in RP-HPLC Fraction
		100	300	600	900	1200	1500	
1	mg TOTAL	5.62	16.86	33.72	50.58	67.44	94.40	KUN-5 [A8Y7N8], RTS-DISI [AUF41658]
	mg RET	4.17	7.06	**9.33**	8.61	8.81	8.00	
2	mg TOTAL	3.08	9.24	18.48	27.72	36.96	46.20	KUN DrKIn-II [H6VC06], KUN-1/5 AFE83617]
	mg RET	1.85	3.83	**5.06**	3.82	4.06	4.21	
3	mg TOTAL	0.23	0.69	1.38	2.07	2.76	3.45	
	mg RET	0.20	0.60	**1.11**	0.89	0.84	0.90	
4	mg TOTAL	1.67	5.01	10.02	15.03	20.04	28.53	KUN DrKIn-II [H6VC06], [A8Y7N6/7]
	mg RET	1.27	2.91	**4.39**	1.81	3.14	0.00	
6	mg TOTAL	7.02	21.06	42.12	63.18	84.24	28.53	VEGF-F [P0DL42]
	mg RET	7.02	21.06	**42.12**	39.85	34.84	33.16	
11	mg TOTAL	26.21	78.63	157.26	235.89	314.52	393.15	PLA2 RV4 [Q02471]
	mg RET	27.70	77.23	**122.74**	105.19	117.07	119.20	
12	mg TOTAL	8.07	24.21	48.42	72.63	96.84	121.05	SVSP RVV-V [P18964]
	mg RET	8.07	24.21	48.42	72.63	**78.92**	73.75	
13	mg TOTAL	26.87	80.61	161.22	241.83	322.44	403.05	PLA2 RV7 [P31100]
	mg RET	27.73	74.73	**116.50**	114.58	107.27	108.89	
14	mg TOTAL	3.32	9.96	19.92	29.88	39.84	49.80	SVSP VLSP-1
	mg RET	3.07	9.21	18.41	27.62	**32.07**	31.76	
15	mg TOTAL	2.45	7.35	14.70	22.05	29.40	36.75	PIV-SVMP RVV-FX
	mg RET	1.55	4.64	9.28	13.91	18.55	**23.19**	
17	mg TOTAL	4.44	13.32	26.64	39.96	53.28	66.60	CTL-4 [Q4PRC9], P68 [ADK22825]
	mg RET	4.44	13.32	26.64	39.96	53.28	**66.60**	
18	mg TOTAL	4.39	13.17	26.34	39.51	52.68	65.85	CTL-4, CTL-3 [4QPRD0], PIV-SVMP RVV-FX
	mg RET	4.39	13.17	26.34	39.51	52.68	**65.85**	
19	mg TOTAL	3.99	11.97	23.94	35.91	47.88	59.85	CTL-4, CTL-3, PIV-SVMP RVV-FX
	mg RET	3.99	11.97	23.94	35.91	47.88	**59.85**	

## Supplementary Materials

The following are available online at http://www.mdpi.com/2414-6366/3/2/66/s1, Table S1: Bottom-up MS/MS identification of proteins fractionated by reverse-phase HPLC and SDS-PAGE from the venom of adult Russell’s viper (*Daboia siamensis*) from Taiwan; Table S2: MS/MS identification of protein spots resolved by 2DE analysis of venom from adult Russell’s viper (*Daboia siamensis*) from Taiwan.
